# A De Novo heterozygous frameshift mutation identified in *BCL11B* causes neurodevelopmental disorder by whole exome sequencing

**DOI:** 10.1002/mgg3.897

**Published:** 2019-07-25

**Authors:** Fengchang Qiao, Chen Wang, Chunyu Luo, Yan Wang, Binbin Shao, Jianxin Tan, Ping Hu, Zhengfeng Xu

**Affiliations:** ^1^ Department of Prenatal Diagnosis Women's Hospital of Nanjing Medical University, Nanjing Maternity and Child Health care Hospital Nanjing China

**Keywords:** *BCL11B*, developmental delay, intellectual disability, neurodevelopment, whole exome sequencing

## Abstract

**Background:**

Next‐generation sequencing has been invaluable to delineate the genetic etiology of neurodevelopmental disorders (NDDs) in recent years. *BCL11B*, encoding Cys_2_His_2_ zinc finger transcription factor, is essential for the development of immune and neural systems.

**Methods:**

Herein, we describe a Chinese girl presenting craniofacial abnormalities, developmental delay and intellectual disability with speech impairment. Exomes of genes were enriched with the Agilent SureSelect QXT ALL Human Exon V6 kit and sequenced on Illumina Hiseq 2500 platform.

**Results:**

After variants filtering and annotation, we identified a de novo heterozygous 11bp frameshift mutation NM_138576.4: c.2190_2200delGGACGCACGAC (p.Thr730Thrfs*151) in exon 4 of *BCL11B*, which is expected to escape nonsense‐mediated mRNA decay and probably result in a truncated protein with lack of the C‐terminal DNA‐binding zinc‐finger domains.

**Conclusion:**

This is the first report of NDD caused by a *BCL11B* variant in a Chinese population. The mutation identified in this report broadens the knowledge of mutation spectrum of *BCL11B* and might help in genetic counseling and reducing reproductive risk.

## 1 INTRODUCTION

Neurodevelopmental disorders (NDDs), which comprise epilepsy, intellectual disability (ID) and autism spectrum disorder, are a highly heterogeneous group of disorders caused by defects in genes implicated in development and function of the nervous system. To date, over 2000 genes have been correlated with ID, and very few occur at high prevalence (Martinez et al., 2017; Vissers, Gilissen, & Veltman, 2016). Considering the high genetic heterogeneity of ID, currently available whole exome sequencing (WES) offers a powerful approach to explore the genetic etiology of ID and identify ID‐related genes. Moreover, trio WES could detect de novo mutations (Veltman & Brunner, 2012), such as small indels and single‐nucleotide variants, which constitute a main contributing factor to the genetic etiology of mild to profound ID and NDDs (Deciphering Developmental Disorders Study, 2017; Hamdan et al., 2014; Rauch et al., 2012; Wilfert, Sulovari, Turner, Coe, & Eichler, 2017).

*BCL11B* (BAF chromatin‐remodeling complex subunit [MIM:606558]) is a kruppel‐like, lineage‐specific Cys_2_His_2_ zinc finger transcription factor, which regulates different physiological processes such as cell proliferation, differentiation, and apoptosis (Lennon, Jones, Lovelace, Guillemin, & Brew, 2017). *BCL11B* is a known modulator of early thymocyte development, and somatic *BCL11B* variants have been involved in a wide range of malignant transformation, including in T‐cell acute lymphoblastic leukemia (Gutierrez et al., 2011; Neumann et al., 2015). *BCL11B* plays a critical role in murine neurogenesis, such as the differentiation of striatal medium spiny neurons (Arlotta, Molyneaux, Jabaudon, Yoshida, & Macklis, 2008), the development and maintenance of the dentate gyrus (Simon et al., 2016, 2012), and the development of corticospinal motor neurons (Arlotta et al., 2005). Until now, there are only two reports on germline *BCL11B* variants and human rare diseases. Punwani et al. (2016) reported a male infant bearing a de novo* BCL11B* missense mutation with severe developmental delay, absence of corpus callosum, craniofacial abnormalities, and severe combined immunodeficiency (SCID). Lessel et al. (2018) reported 13 individuals harboring heterozygous mutations in *BCL11B*, and all analyzed individuals showed ID, developmental delay and the impairment of T‐cell development, but none displayed obvious clinical signs of immune deficiency.

Here, we reported a Chinese girl with neurodevelopmental abnormalities and identified a novel heterozygous frameshift mutation in *BCL11B* through WES.

## 2 MATERIALS AND METHODS

### 2.1 Editorial policies and ethical considerations

This study was approved by the Ethics Committee of the Nanjing Maternity and Child Health Care Hospital and adhered to the tenets of the Declaration of Helsinki. Informed written consent was obtained from the parents of the patient. All authors made substantial contributions to the work including conception and design, acquisition, or interpretation of data and implicated in drafting the manuscript or revised it critically for important intellectual content. All authors approved the final version of manuscript to be published and agreed to be responsible for all aspects of the work in ensuring that questions related to the accuracy or integrity of any part of the work are properly investigated and resolved.

### 2.2 Clinical report

**Figure 1:**
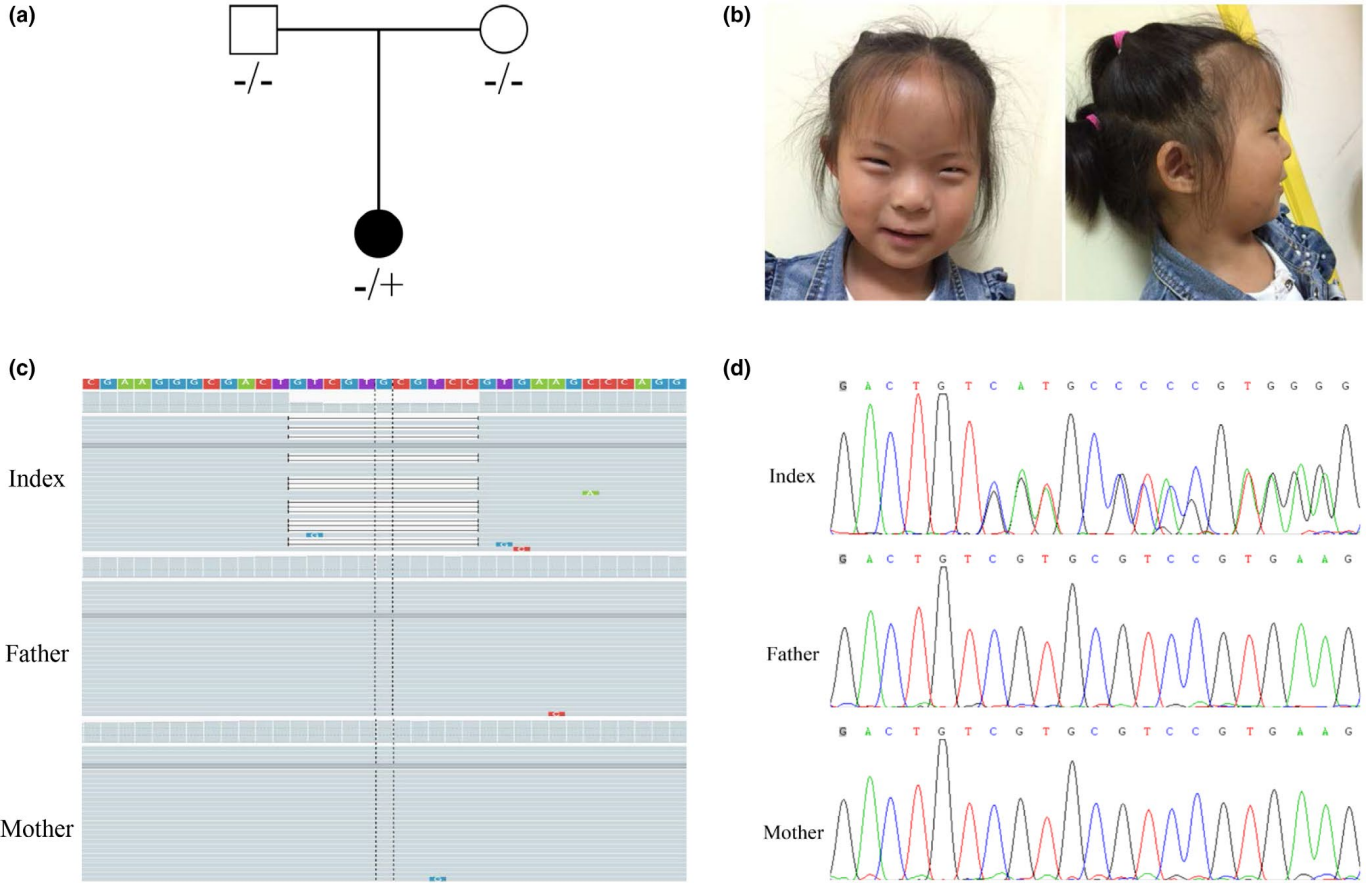
Pedigree and mutation analysis of the family. (a) Pedigree of the family. (b) Clinical features of our patient show facial dysmorphism. (c) Alignment of exome sequences to hg19 reveals a de novo heterozygous 11bp frameshift mutation in exon 4 of *BCL11B*. (d) Sanger sequencing shows that the proband carried a heterozygous frameshift mutation (c.2190_2200delGGACGCACGAC), which was not found in her parents

The female infant was born to healthy nonconsanguineous parents who had no known family history of NDD (Figure 1a). The child was born at 38 weeks of gestation after uneventful pregnancy and a cesarean section. Birth parameters were normal (weight of 3,400 g, head circumference of 34.5 cm, and length of 49.5 cm) and no congenital anomaly was observed. She sat unsupported at 12 months, walked at 25 months of age, and she had an ID (IQ 55) with speech impairment. The girl is subjected to more respiratory infections than usual and she did not require hospitalization and prolonged or extraordinary treatments, therefore hematological assessment of her immune system have not been performed. Her parents first came to our center for genetic counseling when she was 5 years old. At that point, craniofacial abnormalities consisting of micrognathia, hypertelorism, short palpebral fissures, bilateral epicanthi, sparse eyebrows, everted upper part of the ears, small attached ear lobe, and thin upper lip were noted and the remainders of physical examination were normal (Figure 1b). She smiles often and presents good eye contact but lacks expressive or receptive languages. The result of magnetic resonance imaging of brain was normal.

### 2.3 Whole exome sequencing

Genomic DNA of the proband and her parents was extracted from peripheral blood for genetic analysis. WES was conducted on 500 ng of genomic DNA from the proband and her parents. Fragment libraries were created from the sheared samples by sonication and target enrichment was performed according to the manufacturer's protocols (Agilent SureSelect QXT ALL Human Exon V6 kit). Captured DNA was amplified followed by solid‐phase bridge amplification and paired‐end sequenced on Illumina Hiseq 2500 (Illumina, Inc.). Alignment of reads to the human reference sequence (hg19 assembly) and variants detection was performed using Genome Analysis Toolkit 3.4 (GATK, www.broadinstitute.org/gatk). The variant annotation information was obtained from SeattleSeqAnnotation (snp.gs.washington.edu/SeattleSeqAnnotation 138) and novel variants were filtered against 1000 Genomes (1000 genomes release phase 3, http://www.1000genomes.org/), dbSNP (http://www.ncbi.nlm.nih.gov/ projects/SNP/snp_summary.cgi), and Genome Aggregation Database (gnomad.broadinstitute.org). PCR and Sanger sequencing was finally applied to validate the mutation (forward primer 5′‐GCCAAGCGCATCAAGGTG‐3′ and reverse primer 5′‐TACTCGCACGTGTCGCTG‐3′).

## 3 RESULTS

**Figure 2:**
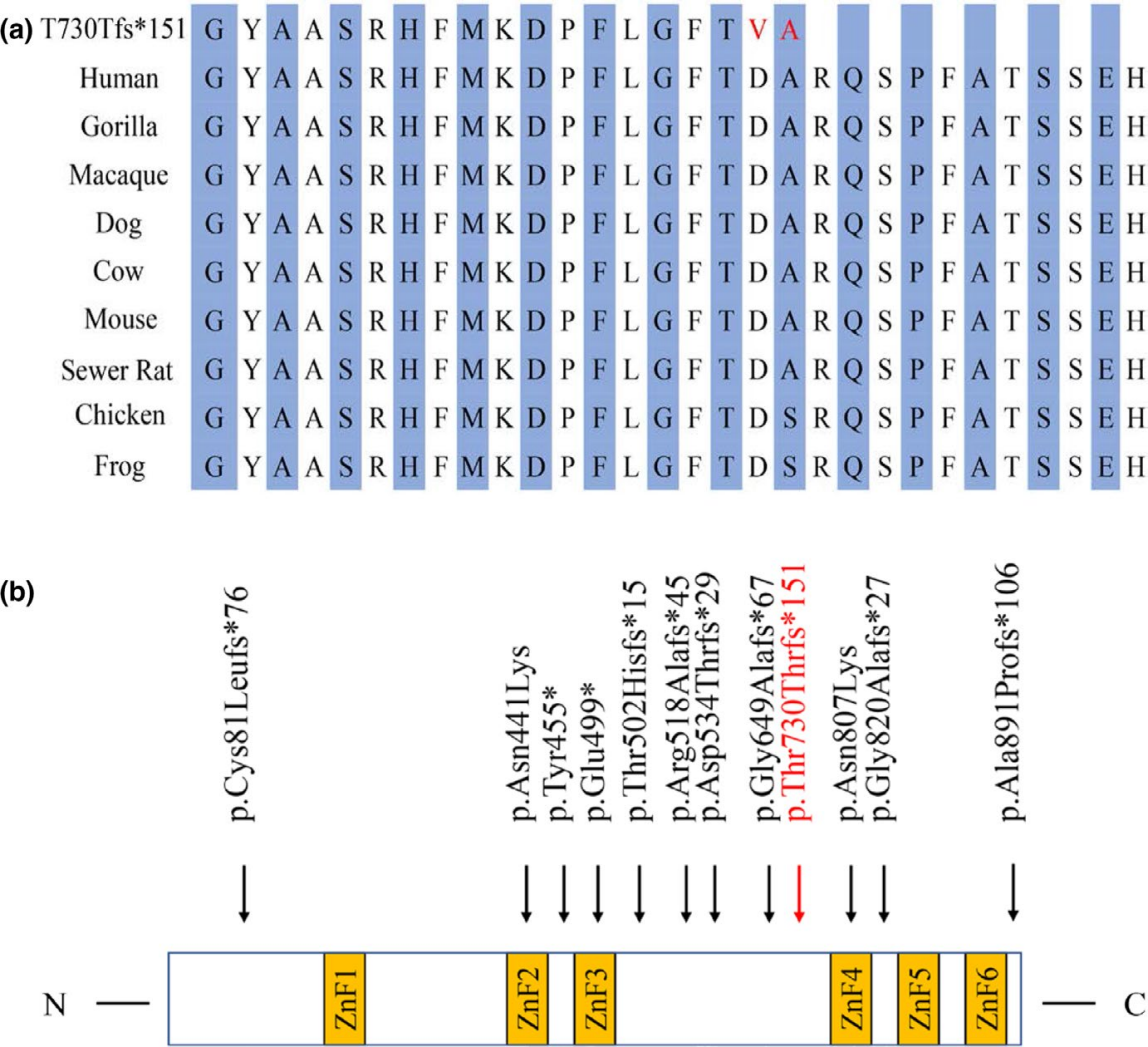
(a) Conserved amino acid sequences of *BCL11B* protein and the predicted truncated *BCL11B* protein caused by the frameshift mutation identified in this proband. (b) Mutation spectrum of *BCL11B* and schematic protein structure of *BCL11B*. The frameshift mutations identified in the proband in this study are marked with vertical arrows and shown in red, and the recently identified variants are shown in black. C, C terminus; N, N terminus; ZnF, zinc‐finger C2H2 domain

Chromosomal microarray analysis did not pinpoint any pathogenic copy number variants. Exome sequencing in the proband produced about 70.71 million reads with a read length of 150 bp. There were 99.55% reads aligned to the human reference genome; 3,908.65 Mb were mapped to the target region with a mean coverage of 98.24×. There were 56,880 SNPs, including 46,863 nonsynonymous SNPs in the coding sequence and 1847 in the splice sites. There were 6,132 indels, including 539 in the coding sequence and 560 in the splice sites. A prioritized filtration strategy of the variants was performed following a guidance to narrow down the potentially causative variants (Roy et al., 2018). An analysis of whole‐exome sequences from the proband and her parents ruled out nonpaternity, the highest priority variant identified by WES analysis in the patient was a de novo heterozygous 11bp frameshift mutation in exon 4 of *BCL11B* (NM_138576.4: c.2190_2200delGGACGCACGAC (p.Thr730Thrfs*151)) (Figure 1c), which is expected to escape nonsense‐mediated mRNA decay and probably results in a truncated protein with lack of the C‐terminal DNA‐binding zinc‐finger domains. MutationTaster software analysis disclosed that the variant could be a disease‐causing variant with a probability value close to 1, suggesting it is highly secure. The variant was further confirmed by Sanger sequencing and the results also showed that the variant arose de novo (Figure 1d). The nucleotide change affects a highly conserved amino acid in the *BCL11B* domain (Figure 2a). The variant was absent from the 1000 Genomes, ExAC, and gnomAD databases. According to the pLI (probability of loss‐of‐function intolerance) value in the ExAC Browser, *BCL11B* seems to be strongly intolerant to heterozygous loss‐of‐function variants (pLI = 0.93) (Lek et al., 2016). Taken together, the identified germline variant was classified as pathogenic according to ACMG guidelines (Richards et al., 2015). A hemizygous variant in *ARX* (NM_139058.2: c.707 A>G (p.Asp236Gly)) and compound heterozygous variants in *VAC14* (NM_018052.3: c.1095 T>G (p.Ser365Arg); c.1327 C>T (p.Leu443Phe)) were also identified, which were ruled out because two of the variants were classified as uncertain of significance (Table S1).

## 4 DISCUSSION

Recently, Punwani et al. (2016) identified a de novo* BCL11B* missense alteration (c.1323T>G, p.Asp441Lys) in a male infant characterized by SCID, lack of corpus callosum, craniofacial abnormalities, and global developmental delay (Table 1). Lessel et al. (2018) described 13 patients harboring heterozygous *BCL11B* mutations, namely one missense mutation (c.2421C>G, p.Asn807Lys), two nonsense mutations (p.Try455*; p.Glu499*), and seven frameshift mutations (p.Cys81Leufs*76; p.Thr502Hisfs*15; p.Arg518Alafs*45; p.Asp534Thrfs*29; p.Gly649Alafs*67; p.Gly820Alafs*27; p.Ala891Profs*106). The affected individuals manifested with mild to moderate ID and language impairment, craniofacial abnormalities, global developmental delay, along with abnormal development of the immune system, but without obvious clinical signs of immune deficiency (Table 1). These variants, as well as the pathogenic variant (p.Thr730Thrfs*151) we identified here, cluster in regions of *BCL11B* that encode the zinc finger motifs. Structural homology modeling of the *BCL11B* variants in T‐cell acute lymphoblastic leukemia indicates that the disruption of zinc finger might impair DNA binding (Gutierrez et al., 2011). Of the 10 identified nonsense and frameshift variants, p.Cys81Leufs*76 and p.Ala891Profs*106 are expected to cause haploinsufficiency, while the others are expected to render a protein with lack of the C‐terminal DNA‐binding zinc‐finger domains (Figure 2b).

**Table 1:** Clinical characteristics of patients with *BCL11B* variants

Case	1	2	3	4	5	6	7	8	9	10	11	12	13	14#
Gender	M	F	M	M	F	M	M	F	M	M	F	M	M	F
Ethnicity	US	Caucasian	Caucasian	Caucasian	Arab	Caucasian	Brazilian	Caucasian	Caucasian	Caucasian	Caucasian	Caucasian	North Africa	China
Variant	p.Asp441 Lys	p.Gly820 Alafs*27	p.Gly649 Alafs*67	p.Ala891 Profs*106	p.Thr502 Hisfs*15	p.Asn807 Lys	p.Cys81 Leufs*76	p.Asp534 Thrfs*29	46,XY, t(4;14) (p15;q32.1)	46,XY, t(4;14) (q31.1;q32.2)	p.Glu499*	p.Tyr455*	p.Arg518 Alafs*45	p.Thr730 Thrfs*151
Intellectual disability	+	+	+	+	+	+	+	+	+	+	+	+	+	+
Speech impairment	+	+	+	+	+	+	+	+	+	+	+	+	+	+
Delay in motor development	+	+	+	+	+	+	+	+	+	−	+	+	+	+
Autistic features	−	+	−	+	−	−	−	+	+	−	−	−	−	−
Myopathic facial appearance	−	+	−	+	+	+	−	+	−	−	+	−	−	−
Thin eyebrows	+	+	+	+	−	−	−	+	−	−	−	−	+	+
Small palpebral fissures	+	+	+	+	−	+	−	+	−	+	+	−	−	+
Hypertelorism	+	+	−	+	+	+	−	+	−	−	−	+	+	+
Prominent nose	+	+	+	+	−	+	+	+	+	+	−	−	+	+
Long philtrum	+	+	+	+	+	−	−	+	−	+	+	+	+	+
Thin upper lip	+	+	+	+	+	+	+	+	+	+	−	+	+	+
Refractive error	−	Hyperopia	−	Hyperopia	−	−	Myopia	−	−	−	Myopia	Exotropia	−	−
Dental anomalies	+	+	+	−	−	+	+	−	−	−	−	−	+	−
Feeding difficulties	−	−	−	+	−	+	−	−	−	−	−	+	−	−
Immune response	Low TREC at birth	−	Frequent infections	Frequent infections	−	Low TREC at birth	Frequent/atypical infections	−	−	−	−	−	Frequent infections	Frequent infections
Allergy/asthma	+	−	−	+	−	+	+	−	+	−	+	+	+	−

Note

“+” represents present; “−” represents absent; TREC, T‐cell receptor excision circles; #, The patient in our study.

Recent reports manifested that patients with a SCID (Lessel et al., 2018; Punwani et al., 2016), both harboring missense alterations in *BCL11B*, were more seriously affected than other patients with nonsense/frameshift mutations. Remarkably, the ZnF_C2H2 specificity residues of the DNA‐interacting alpha helix were perturbed by these missense variants. Punwani et al. (2016) used ChIP‐seq analysis to reveal that the p.Asn441Lys substitution caused not only decreased *BCL11B* binding to canonical target DNA sites but also induced binding to new DNA sites. As *BCL11B* acts as either a transcriptional repressor or an activator depending on its posttranslational modifications (Kominami, 2012), the differential binding to new sites might account for the observed phenotypic differences. Nevertheless, we cannot preclude the other pathogenic variants that were either not appropriately captured by WES or are hidden in intergenic or deep intronic regions. Hence, we anticipate that additional patients carrying missense alterations in ZnF_C2H2 domains will be identified, which is necessary to elucidate the phenotypic spectrum and their possible mechanisms.

Taken together, we reveal that the variant (p.Thr730Thrfs*151) leading to a truncation of the *BCL11B* protein mostly causes NDD, underlining an important role for *BCL11B* in the development of the human neural systems. This is the first report of NDD caused by a *BCL11B* variant in a Chinese population. The mutation identified in this report broadens the knowledge of mutation spectrum of *BCL11B* and might help in genetic counseling and reducing reproductive risk.

## CONFLICT OF INTEREST

The authors declare that they have no conflict of interest.
