# Supplementary material for: A De Novo heterozygous frameshift mutation identified in BCL11B causes neurodevelopmental disorder by whole exome sequencing
Source: Mol Genet Genomic Med. 2019 Jul 25;7(9):e897. doi: 10.1002/mgg3.897 (PMC6732278; doi:10.1002/mgg3.897)
Supplement: Supplementary file 1 [file MGG3-7-e897-s001.docx]

**Table 1** High priority variants in the proband from exome sequence analysis

| Gene | NM number | Variant | Zygosity | Inheritance | Disease Phenotype | ACMG classification |
| --- | --- | --- | --- | --- | --- | --- |
| ARX | NM_139058.2 | c.707 A>G(p.Asp236Gly) | Het | XD | Hydranencephaly with abnormal genitalia  Lissencephaly, X-linked 2  Proud syndrome | Uncertain of significance |
| VAC14 | NM_018052.3 | c.1095 T>G(p.Ser365Arg) | Het | AR | Striatonigral degeneration, childhood-onset | Uncertain of significance |
| VAC14 | NM_018052.3 | c.1327 C>T(p.Leu443Phe) | Het | AR | Striatonigral degeneration, childhood-onset | Likely pathogenic |
